# Supplementary material for: A mouse model of ATRX deficiency with cognitive deficits and autistic traits
Source: J Neurodev Disord. 2023 Nov 13;15:39. doi: 10.1186/s11689-023-09508-7 (PMC10644498; doi:10.1186/s11689-023-09508-7)
Supplement: Supplementary file 1 — Additional file 1: Fig. S1. Validation of ATRX deletion in AtrxNEXCre female mice. A) ATRX immunofluorescence staining (red) of E13.5 control and AtrxNEXCre brain coronal cryosections. DAPI counterstaining is shown in blue. Scale bar, 570 μm. B) Magnified view of ATRX (top, red) and βIII-tubulin (bottom, green) staining in the developing cortical plate. Scale bar, 144 μm. C) ATRX (red) and DAPI (blue) staining in the cortex and hippocampus of AtrxNEXCre and control mice at P20. D) ATRX (red) and NeuN (green) staining in the cortex and hippocampus at P20. Cortex scale bar,100 μm and hippocampus scale bar, 400 μm. CA: cornu Ammonis, CP: cortical plate, Ctx: cortex, DG: dentate gyrus, GE: ganglionic eminence, LV: lateral ventricle, VZ: ventricular zone. eminence. Magnified images of cortical plate scale bar, 144 μm. VZ: ventricular zone, CP: cortical plate. Fig. S2. Spatial learning deficits in AtrxNexCre mice. A) Latency to find the platform during the training phase of the Morris water maze test shows that AtrxNEXCre male and female mice have impaired spatial learning (p<0.0001, F=3.630). B) AtrxNexCre mice spend less time swimming during the trials, reflecting their floating behaviour (p<0.0001, F=19.50) (Controlmale n=7, AtrxNEXCre male n=7, Controlfemale n=7, AtrxNEXCre female n=7). C) A 6-minute forced swim test revealed increased apathy in water for male mice (p=0.0292, F=2.028) (Controlmale n=15, AtrxNEXCre male n=12) and decreased apathy in female mice (p=0.0154, F=3.149, Controlfemale n=13, AtrxNEXCre female n=12). Sex discrimination index reveals sex differences in the forced swim test (p=0.0002, F=2.370). Error bars represent +/-SEM. Three-way ANOVA or Students t-test performed when appropriate (* p<0.05, ** p<0.001, ***p<0.0001). Fig. S3. Self injury and reduced olfaction in AtrxNEXCre mice. A) Example images of mice with evidence of self-injury caused by over-grooming. B) Male and female AtrxNEXCre mice spend less time sniffing the almond and banana odors [file 11689_2023_9508_MOESM1_ESM.pdf]

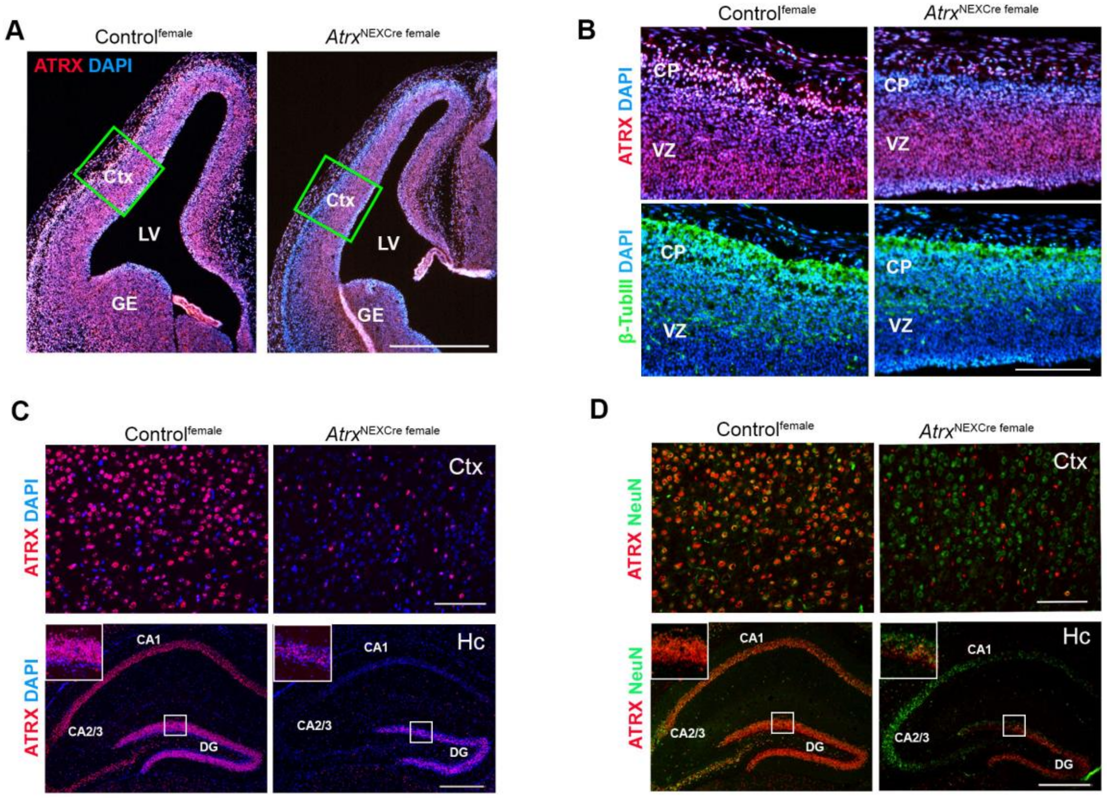

Figure S1: Validation of ATRX deletion in *Atrx*^{NEXCre} female mice.
A) ATRX immunofluorescence staining (red) of E13.5 control and *Atrx*^{NEXCre} brain coronal cryosections. DAPI counterstaining is shown in blue. Scale bar, 570 µm. **B)** Magnified view of ATRX (top, red) and β III-tubulin (bottom, green) staining in the developing cortical plate. Scale bar, 144 µm. **C)** ATRX (red) and DAPI (blue) staining in the cortex and hippocampus of *Atrx*^{NEXCre} and control mice at P20. **D)** ATRX (red) and NeuN (green) staining in the cortex and hippocampus at P20. Cortex scale bar,100 µm and hippocampus scale bar, 400 µm. CA: cornu Ammonis, CP: cortical plate, Ctx: cortex, DG: dentate gyrus, GE: ganglionic eminence, LV: lateral ventricle, VZ: ventricular zone. eminence. Magnified images of cortical plate scale bar, 144 µm. VZ: ventricular zone, CP: cortical plate.

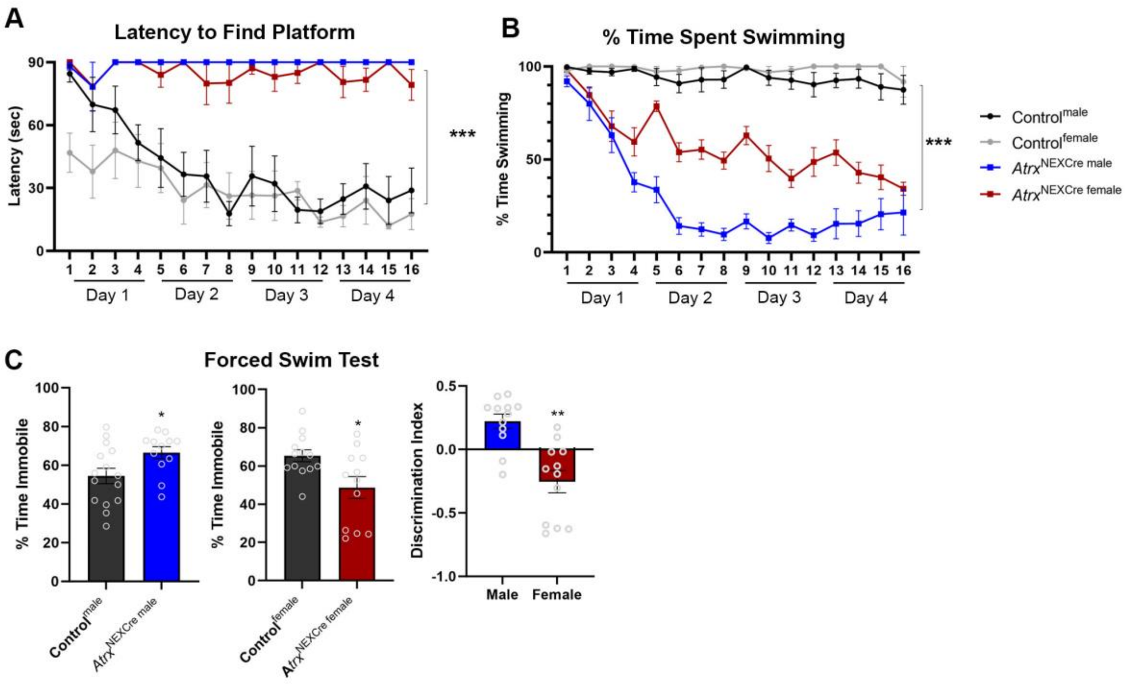

Figure S2: Spatial learning deficits in *Atrx*$^{\mathbf{NexCre}}$ mice

A) Latency to find the platform during the training phase of the Morris water maze test shows that *Atrx*^{NEXCre} male and female mice have impaired spatial learning ($p<0.0001$, $F=3.630$). **B)** *Atrx*^{NexCre} mice spend less time swimming during the trials, reflecting their floating behaviour ($p<0.0001$, $F=19.50$) (Control^{male} n=7, *Atrx*$^{NEXCre\ male}$ n=7, Control^{female} n=7, *Atrx*^{NEXCre} female n=7). **C)** A 6-minute forced swim test revealed increased apathy in water for males ($p=0.0292$, $F=2.028$)(Control^{male} n=15, *Atrx*$^{NEXCre\ male}$ n=12) and decreased apathy in females ($p=0.0154$, $F=3.149$, Control^{female} n=13, *Atrx*$^{NEXCre\ female}$ n=12). Sex discrimination index indicates sex differences in the forced swim test ($p=0.0002$, $F=2.370$). Error bars represent +/-SEM. Three-way ANOVA or Students t-test performed when appropriate (* $p<0.05$, ** $p<0.001$, *** $p<0.0001$).

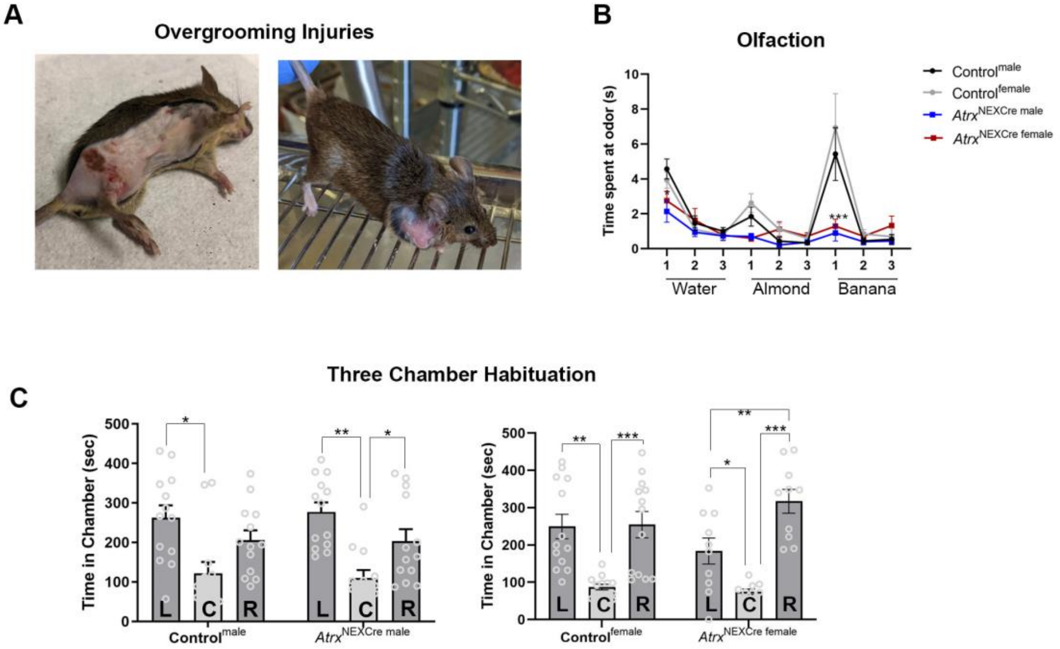

Figure S3: Self injury and reduced olfaction in *Atrx*^{NEXCre} mice.

A) Example images of mice with evidence of self-injury caused by over-grooming. **B)** Male and female *Atrx*^{NEXCre} mice spend less time sniffing the almond and banana odors during the olfaction test ($p<0.0001$, $F=18.65$; Control^{male} n=17, *Atrx*$^{NEXCre\ male}$ n=14, Control^{female} n=13, *Atrx*$^{NEXCre\ female}$ n=13). **C)** Male and female mice of each genotype explored the three-chamber apparatus freely during the 10-minute habituation portion of the three-chamber assay (Control^{male}: n=13, *Atrx*$^{NEXCre\ male}$ n=13, Control^{female}: n=13, *Atrx*$^{NEXCre\ female}$ n=11) (L= left side, C= center, R= right side). Error bars represent +/-SEM. Two-way Anova with Sidak post-hoc (*$p<0.05$, ** $p<0.001$, ***$p<0.0001$).
